# Hyaluronic acid as a biomaterial for dental pulp regeneration: a systematic review of preclinical studies

**DOI:** 10.3389/fbioe.2025.1661188

**Published:** 2025-09-05

**Authors:** Azal Hadi Al-Masoody, Mahshid Hodjat, Tahereh Sadat Jafarzadeh Kashi

**Affiliations:** ^1^ Department of Dental Biomaterials, Faculty of Dentistry, Tehran University of Medical Sciences, Tehran, Iran; ^2^ College of Dentistry, University of Alkafeel, Najaf, Iraq; ^3^ Dental Research Centre, Dentistry Research Institute, Tehran University of Medical Sciences, Tehran, Iran

**Keywords:** hyaluronic acid, dental pulp, endodontic regeneration, dentinogenesis, vital pulp therapy (VPT)

## Abstract

**Introduction:**

With the current advancements in regenerative medicine, it has become necessary to refine the current regenerative endodontic procedure (REP). Scaffold improvement, including the use of hyaluronic acid as a natural biomaterial, has been the subject of many studies. This systematic review aims to explore the effects of hyaluronic acid (HA) on dental pulp regeneration.

**Methods:**

A comprehensive search on Hinari, PubMed, Springer, and ScienceDirect databases in July 2024 was carried out. All *in vitro* and animal studies that assessed the effects of HA on cell vitality, proliferation, dentinogenesis, neovascularisation and neurogenesis in an endodontic context were included. Clinical studies were excluded. All articles were screened and assessed for relevance by the authors. The quality and risk of bias of the included studies were evaluated using the QUIN, SYRCLE, and ARRIVE Essential 10 tools.

**Results:**

A total of 23 articles were included, comprising 17 *in-vitro*, 5 animal, and 1 combined *in vitro* and animal study. Thematic synthesis of results was adopted. The methodologies for HA addition, HA concentration and molecular weight were different across the articles. Most *in-vitro* studies showed that HA have a neutral effect on cell proliferation, and a positive effect on dentinogenesis and neovascularisation. Most animal studies showed increased dentine bridge formation.

**Discussion:**

The variability in the study design has made it difficult to assert the results; however, most studies agree that HA has promising potential in REP.

**Systematic Review Registration:**

[https://osf.io/t45ec/].

## 1 Introduction

### 1.1 Dental pulp regeneration

Tooth loss from periodontal disease, trauma, or caries has a negative impact on general health and oral hygiene (Saintrain and De Souza, 2012). Root canal therapy (RCT), which involves the removal of pulp tissue followed by cleaning and obturating the root canal system, has long been the standard treatment method for necrotic or inflamed dental pulp. While RCT is effective in removing infection, it leaves the tooth unviable, resulting in a weakened structure and an increased risk of fracture (Plotino et al., 2017). As a promising alternative, regenerative endodontic therapy (REP) aims to induce dentinogenesis and pulpal revascularisation, thus preserving the tooth vitality and structure (Kontakiotis et al., 2015). The American Association of Endodontists and the European Society of Endodontology have released REP protocols with mostly similar procedures (Kahler et al., 2024). Besides REPs, vital pulp therapies like pulp capping and pulpotomies also fall within the area of dental pulp regeneration as they attempt to regenerate part of the dental pulp (Murray, 2023).

Pulp regeneration requires the presence of three components: scaffold, stem cells, and signalling molecules. In the current protocols, the blood clot, that forms in dental canals after bleeding is provoked, plays the role of the scaffold containing signalling molecules, and might contain stem cells as well is typically a blood clot (Kontakiotis et al., 2015). However, due to the uncertainty in the success of this procedure, researchers have been investigating ways to enhance these components of the REP. Scaffolds of diverse types, including natural, synthetic, and combination scaffolds, have been studied to improve the outcomes of pulp regeneration (Liu et al., 2022; Li et al., 2024). For a scaffold to be effective, it must be bioactive, biodegradable, structurally stable, easily injected into the typically complicated shape of the dental canals, and preferably have antimicrobial activity. However, caution must be exercised as overstimulation of dentinogenesis could lead to negative results, such as radicular canal obliteration or pulp stone formation (Kim, 2016). The regenerative process must, therefore, be carefully balanced to avoid such complications. Despite extensive research, the perfect scaffold that provides all the mentioned properties is yet to be discovered.

Regenerating dental pulp involves a complex series of processes. It comprises several biological activities, including the release of molecular signals, stem cell homing, and the formation of new neurons, blood vessels, odontoblasts, and loose connective tissue (Retana-Lobo and Retana-Lobo, 2018; Li et al., 2024). Several intercellular molecular signals are involved in endodontic regeneration to induce cellular chemotaxis and differentiation (Smith et al., 2016). Given that the extracellular matrix of the dentin/pulp complex is composed of collagen and non-collagenous proteins, it has been common for researchers to examine the presence of these proteins as a marker for dental pulp regeneration. (Hong et al., 2019; Noohi et al., 2023). Additionally, the presence of vascular and neural growth factors, along with the presence of dentin/pulp proteins, is further investigated to examine the efficacy of different scaffold designs (Ahmed et al., 2021).

### 1.2 Hyaluronic acid in regenerative endodontic procedure

Hyaluronic acid (HA) is a biocompatible, naturally occurring glycosaminoglycan that is the key component of the extracellular matrix. It has been proposed as a potential replacement for blood clot replacement in REP. It constitutes the majority of the vitreous body of the eye, and half the human body’s HA is found in the skin (Papakonstantinou et al., 2012). HA has excellent scaffolding properties and plays a crucial role in infection protection and wound healing (Ahmadian et al., 2019). It can be derived from either natural or synthetic sources and can be prepared in various forms, such as liquids, hydrogels, or sponges. Additionally, HA can be mixed with other materials to enhance specific properties (Inuyama et al., 2010).

Many researchers have investigated the potential of HA in dental pulp regeneration (Miglani et al., 2023). *In vitro* models have been used to evaluate the effects of HA on stem cell migration, proliferation and differentiation, simulating the biological process that happens during dental pulp regeneration. Meanwhile, *in vivo* models examined the impact of HA and HA-based scaffolds on tooth revitalisation and the related histological changes in the pulp. Casale et al., in (2016), have conducted a systematic review about the uses of hyaluronic acid as an adjuvant treatment for inflammatory diseases in the oral cavity. Previous systematic reviews have searched the effectiveness of HA as an adjuvant treatment for oral inflammatory diseases (Casale et al., 2016; Onisor et al., 2022). Others have discussed the HA effects in bone regeneration (Lorenzi et al., 2024) and treatment for arthritis (Arrich et al., 2005; Altman et al., 2015) and for temporomandibular joint disorders. To our knowledge, no systematic review has been published discussing the effects of HA on dental pulp regeneration until now.

### 1.3 Aim

This review explores all available studies investigating HA’s role in endodontic regeneration using both *in vitro* and *in vivo* preclinically. The methodologies employed in these studies will be critically analysed to provide a clearer understanding of HA’s potential in regenerative endodontics. Also, this review aims to guide future research by identifying existing knowledge gaps in the field.

## 2 Materials and methods

### 2.1 Search strategy

This systematic review was registered at the Open Science Framework (OSF) (DOI: 10.17605/OSF.IO/T45EC). The research question was: (What is the efficacy of hyaluronic acid as a biomaterial in promoting dental pulp regeneration in preclinical models?). The PICOS framework was adopted as the inclusion criteria before the start of the search process. The PICOS framework for this review was established as follows.

• Population (P): studies investigating dental pulp regeneration procedures, in animal models and *in vitro* settings.
• Intervention (I): Application of HA.
• Comparison (C): No intervention or standard intervention as a control group.
• Outcome (O): 1. Animal studies: Evidence of dental pulp regeneration through clinical, radiographic or histological findings. 2. *In vitro* studies: Assessment of cellular proliferation or differentiation.
• Study design (S): Animal studies and *in vitro* studies.

### 2.2 Study selection and data synthesis

Complying with the PRISMA 2020 guidelines (Page et al., 2021), a comprehensive search was conducted over Hinari, PubMed, Springer, and ScienceDirect databases in July 2024. The following terms were utilised: Hyaluronic Acid, Hyaluronan, Dental Pulp, Regenerative Endodontics, Neovascularisation, Neurogenesis, Cell Movement, and pulp regeneration. Boolean operators were used to join the search words. MeSH terms were used in the PubMed search. The exact search words are shown in Table 1. In the Hinari, Springer, and ScienceDirect databases, the option research article was selected. The duplicate articles have been removed. A thorough search was carried out in the references of review articles. Two authors (AA and MH) independently searched for published studies and assessed the quality of the articles. When there was a disagreement between the researchers, a third researcher intervened (TJ). Animal and laboratory studies were included. Randomised control trials, cohort studies, case-control studies, case series, review articles, case reports, ongoing trials, and retracted articles were excluded. Human studies were excluded to maintain a focused synthesis on the mechanistic evidence derived from preclinical models. All articles that assessed the bone regeneration potential of HA have been excluded since it is out of the scope of this review. Articles that were not indexed in Scopus were excluded. *In-vitro* studies that did not contain an HA-free control group were also excluded. Animal studies that did not contain a control group (HA-free or standard intervention) were excluded.

**TABLE 1:** Search words of the systematic review using different databases.

	Database	Search words
1	PubMed	(“Hyaluronic Acid” [Mesh]) AND (“Dental Pulp” [Mesh]) AND (“Odontogenesis” [Mesh] OR “Regenerative Endodontics” [Mesh] OR “Neovascularization, Physiologic” [Mesh] OR “Neurogenesis” [Mesh] OR “Cell Movement” [Mesh] OR “pulp regeneration”)
2	Springer, ScienceDirect and Hinari	(“Hyaluronic Acid” OR “Hyaluronan”) AND (“Dental Pulp”) AND (“Regenerative Endodontics” OR “Neovascularization” OR “Neurogenesis” OR “Cell Movement” OR “pulp regeneration”)

Thematic synthesis of results was adopted. Results were discussed according to the effectiveness of HA in different aspects of dental pulp regeneration. Each aspect was further analysed using tables, and studies were further explained and compared to each other. Tables were designed to show how the included articles examined the role of HA in cell viability and proliferation, dentin regeneration, revascularisation, and neurogenesis. Concentrations and results of the experiments were also included in the tables.

### 2.3 Risk of bias and quality assessment

#### 2.3.1 *In-vitro* studies

Quality Assessment Tool For *In Vitro* Studies conducted in dentistry (QUIN) tool was implemented to check the quality and the bias risk of the included *in-vitro* studies (Sheth et al., 2024). If the article fulfilled the QUIN criteria, it was given two scores for each criterion. If it was partially fulfilled, it was given one score. A score of zero was given if the article did not fulfil the point. The sum of all scores was then multiplied by 100 and divided by double the number of applicable criteria.

$$\text{Risk of bias}=\frac{\text{sum}\;\text{of}\;\text{scores}*100}{\text{number}\;\text{of}\;\text{applicable}\;\text{criteria}*2}$$

The result measures the risk of bias; less than 50% high risk, 50%–70% medium risk, and more than 70% low risk.

#### 2.3.2 Animal studies

To assess the quality of the articles, the ARRIVE guidelines’ essential 10 were utilised (Percie Du Sertid et al., 2020). If the requirement is mentioned or not mentioned in the article, it is designated as (reported, not reported); if the requirement is partially mentioned, it is designated as (unclear) (Alonso-Fernández et al., 2023). To assess the risk of bias in included animal studies, SYRCLE’s RoB tool was used (Hooijmans et al., 2014; Ma et al., 2020).

## 3 Results

### 3.1 Study selection

**FIGURE 1:**
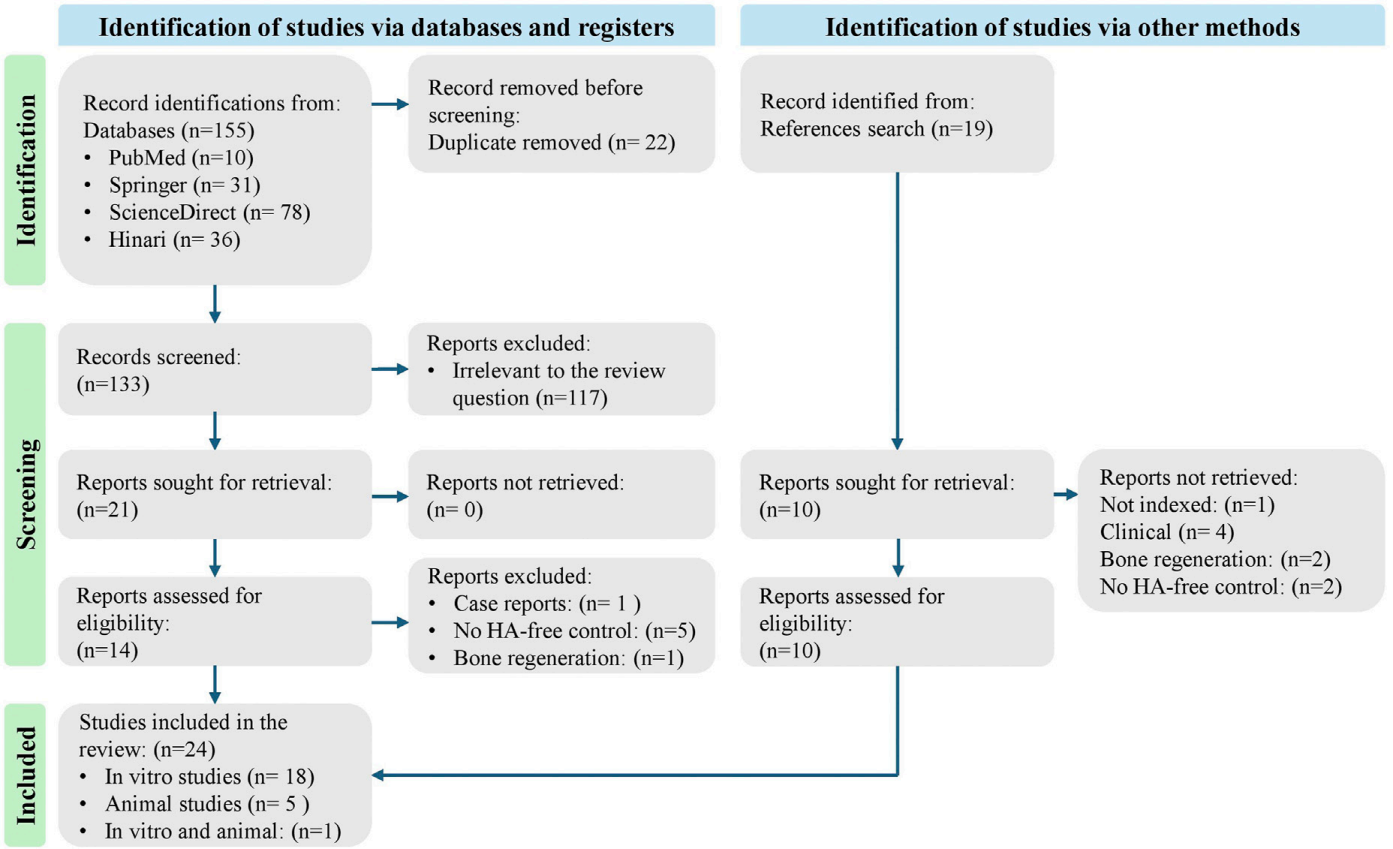
Flowchart of literature data processing according to PRISMA guidelines.

A total of 155 articles were found across all databases. Duplicates were removed, and 133 articles remained. Relevant articles were then selected based on their titles and abstracts, resulting in 20 articles related to the subject of review. Following the exclusion of studies that were not in line with the review question, 13 articles remained. Also, the references for related review articles were thoroughly examined, yielding 18 more articles. Of these, one was not indexed in Scopus, four were clinical studies, two were discussing bone regeneration, and two were *in vitro* studies without an HA-free control group. The final selection consisted of 22 articles: 17 *in-vitro* studies, four animal studies, and 1 combined *in-vitro* and animal study. The identification and screening of articles are shown in the PRISMA flow chart in Figure 1.

The *in vitro* reviewed articles examined the effects of pure or modified HA on dental pulp regeneration by assessing cell morphology, viability, migration, proliferation, dentinogenic differentiation, neovascularisation, and neurogenesis—key factors in endodontic regeneration (Table 2).

**TABLE 2:** Included articles that tested the effects of HA *in vitro*, along with their relevant measurements.

	Author/Year	Relevant measurement^a^
1	Tziafas et al. (1988)	Cellular shape
2	Inuyama et al. (2010)	Cellular differentiation induction
3	Coimbra et al. (2011)	Cell viability
4	Lambricht et al. (2014)	Cell viability
5	Chen et al. (2016)	Dentinogenic
6	Umemura et al. (2016)	Cell proliferation
		Cellular differentiation induction by CD44 expression
		Cellular dentinogenic differentiation
7	Yang et al. (2016)	Cell proliferation
8	Chrepa et al. (2017)	Cell viability, dentinogenesis
9	Cvikl et al. (2017)	Cell viability
10	Niloy et al. (2020)	Cell viability
		Stemness of DPSCs
11	Schmidt et al. (2021)	Cell viability, morphology
		Cell surface marker expression
		Angiogenic gene expression
12	Silva et al. (2021)	Response of HUVEC to the hydrogels
		Angiogenic differentiation of HUVEC
		Migration of endothelial cells
13	Kim et al. (2022)	Cell viability
14	AlHowaish et al. (2022a)	Cell viability
		Dentinogenesis
15	Atila et al./(2022)	Cell viability
		Dentinogenesis
		Angiogenesis
16	Bagio et al. (2023)	Dentinogenesis
		Angiogenesis
17	Nugraheni et al. (2023)	Dentinogenesis
18	Schmidt et al. (2023)	Cell viability, morphology, and adhesion
19	Dorterler et al. (2024)	Cell viability
		Antimicrobial activity

^a^ Only measurements that met the criteria of this systematic review are shown in this table. DPSCs: dental pulp stem cells. HUVEC: human umbilical vein endothelial cells.

### 3.2 Risk of bias and quality assessment

#### 3.2.1 *In-vitro* studies

Most of the articles scored medium in the QUIN tool assessment (Table 3). Two Articles scored low risk of bias, and one scored high risk of bias. No article mentioned how they calculated the sample size, or how they performed randomisation and blinding. Few have mentioned the expertise of the operators and assessors.

**TABLE 3:** Results of QUIN tool assessment of the (included *in vitro* articles).

	Criteria	1. Clearly stated aims/objectives	2. Detailed explanation of sample size calculation	3. Detailed explanation of sampling technique	4. Details of comparison group	5. Detailed explanation of methodology	6. Operator details	7. Randomisation	8. Methods of measurement of outcome	9. Outcome assessor details	10. Blinding	11. Statistical analysis	12. Presentation of results	Final score	Risk of bias
1	Tziafas et al. (1988)	2	0	n/a	2	2	0	0	2	0	0	2	2	54.54545	Medium
2	Inuyama et al. (2010)/*in vitro* part	2	0	n/a	2	2	0	0	2	0	0	2	2	54.54545	Medium
3	Coimbra et al. (2011)	2	0	n/a	2	2	0	0	2	0	0	1	2	50	Medium
4	Lambricht et al. (2014)	2	0	n/a	2	2	0	0	2	0	0	2	2	54.54545	Medium
5	Chen et al. (2016)	2	0	n/a	2	2	0	0	2	0	0	2	2	54.54545	Medium
6	Umemura et al. (2016)	2	0	n/a	2	2	2	0	2	2	0	2	2	72.72727	Low
7	Yang et al. (2016)	2	0	n/a	2	2	2	0	2	0	0	0	2	54.54545455	Medium
8	Chrepa et al. (2017)	2	0	n/a	2	2	0	0	2	0	0	2	2	54.54545	Medium
9	Cvikl et al. (2017)	2	0	n/a	2	2	1	0	2	1	0	2	2	63.63636	Medium
10	Niloy et al. (2020)	1	0	n/a	2	2	0	0	2	0	0	1	2	45.45455	High
11	Schmidt et al. (2021)	2	0	n/a	2	2	1	0	2	1	0	2	2	63.63636	Medium
12	Silva et al. (2021)	2	0	n/a	2	2	2	0	2	2	0	2	2	72.72727	Low
13	Kim et al. (2022)	2	0	n/a	2	2	1	0	2	1	0	2	2	63.63636	Medium
14	AlHowaish et al. (2022a)	2	0	n/a	2	2	1	0	2	1	0	2	2	63.63636	Medium
15	Atila et al. (2022)	2	0	n/a	2	2	1	0	2	1	0	2	2	63.63636	Medium
16	Bagio et al. (2023)	2	0	n/a	2	2	0	0	2	0	0	2	2	54.54545	Medium
17	Nugraheni et al. (2023)	2	0	n/a	2	2	1	0	2	1	0	2	2	63.63636	Medium
18	Schmidt et al. (2023)	2	0	n/a	2	2	0	0	2	0	0	2	2	54.54545	Medium
19	Dorterler et al. (2024)	2	0	n/a	2	2	1	0	2	1	0	2	2	63.63636	Medium

#### 3.2.2 Animal studies

The quality of the included animal studies is listed in Table 4. Only AlHowaish et al. (2022b) fully reported the ARRIVE Essential 10 in their article. Other studies failed to mention the randomisation or the blinding procedures. When subjected to the SYRCLE assessment tool, most questions were answered with (yes), displaying a low RoB. All studies provided insufficient details regarding questions III and V (Table 5). All the points were unclear in the Sasaki and Kawamata-Kido (1995) article, while Palma et al. (2017) presented the highest low RoB scores.

**TABLE 4:** Results of the Quality assessment of the included animal studies using (ARRIVE Essential 10) (Percie Du Sertid et al., 2020).

ARRIVE essential 10		1	2	3	4	5	6
		Sasaki et al. (1995)	Inuyama et al. (2010)	Palma et al. (2017)	AlHowaish et al. (2022b)	Hadi et al. (2023)	Bektas et al. (2024)
I	Study design	Reported	Reported	Reported	Reported	Reported	Reported
II	Sample size	Not reported	Reported	Reported	Reported	Reported	Reported
III	Inclusion and exclusion criteria	Not reported	Reported	Reported	Reported	Reported	Reported
IV	Randomization	Not reported	Not reported	Reported	Reported	Reported	Not reported
V	Blinding	Not reported	Reported	Reported	Reported	Not reported	Reported
VI	Outcome measures	Reported	Reported	Reported	Reported	Reported	Reported
VII	Statistical methods	Not reported	Reported	Reported	Reported	Reported	Reported
VIII	Experimental animals	Reported	Reported	Reported	Reported	Reported	Reported
IX	Experimental procedures	Reported	Reported	Reported	Reported	Reported	Reported
X	Results	Not reported	Reported	Reported	Reported	Reported	Reported

**TABLE 5:** Result of risk of bias assessment (RoB) of the included animal studies using the SYRCLE assessment tool^a^.

Item	Type of bias	SYRCLE domains and judgments	1	2	3	4	5	6
			Sasaki et al. (1995)	Inuyama et al. (2010)	Palma et al. (2017)	AlHowaish et al. (2022b)	Hadi et al. (2023)	Bektas et al. (2024)
I	Selection bias	Sequence generation: Was the allocation sequence adequately generated and applied?	Unclear	Unclear	Yes	Yes	Yes	Unclear
II	Selection bias	Baseline characteristics: Were the groups similar at baseline, or were they adjusted for confounders in the analysis?	Unclear	Yes	Yes	Yes	Yes	Yes
III	Selection bias	Allocation concealment: Was the allocation adequately concealed?	Unclear	Unclear	Unclear	Unclear	Unclear	Unclear
IV	Performance bias	Random housing: Were the animals randomly housed during the experiment?	Unclear	Unclear	Unclear	Unclear	Yes	Unclear
V	Performance bias	Blinding: Were the caregivers and/or investigators blinded from knowledge of which intervention each animal received during the experiment?	Unclear	Unclear	Unclear	Unclear	Unclear	Unclear
VI	Detection bias	Random outcome assessment: Were animals selected at random for outcome assessment?	Unclear	Unclear	Yes	Yes	Unclear	Unclear
VII	Detection bias	Blinding: Was the outcome assessor blinded?	Unclear	Unclear	Yes	Yes	Unclear	Unclear
VIII	Attrition bias	Incomplete outcome data: Were incomplete outcome data adequately addressed?	Unclear	Yes	Yes	Unclear	Unclear	Unclear
IX	Reporting bias	Selective outcome reporting: Are reports of the study free of selective outcome reporting?	Unclear	Yes	Yes	Yes	Yes	Yes
X	Other	Other sources of bias: Was the study apparently free of other problems that could result in a high risk of bias?	Unclear	Yes	Yes	Yes	Yes	Yes

^a^ No, high RoB; Yes, low RoB; Unclear, unclear RoB.

## 4 Discussion

### 4.1 Effects of HA properties on dental pulp regeneration, *in vitro*

#### 4.1.1 HA form and source

Researchers in the included articles selected various sources and forms of HA for the purpose of dental pulp regeneration (Table 6). Most of the studies used HA in liquid form by dissolving sodium hyaluronate salt in water or phosphate-buffered saline. A few studies have used HA as a dermal filler, one as an HA sponge, and another utilised Gengigel gel.

**TABLE 6:** Effects of hyaluronic acid on cellular viability, stemness, proliferation and migration in *in-vitro* studies.

	Author/Year	Cell type	HA form/Source/MW	HA concentration	Cell viability, proliferation and/or migration assays	Findings
1	Tziafas et al. (1988)	Dental papillae of mouse embryos	HA from bovine vitreous humour (grade IV) containing 3.3% Na and 0.025% K/ Sigma/ Not mentioned	0.1, 0.2, and 0.4 mg/mL HA in culture medium	Histological analysis	maintenance of the post-mitotic, polarised cells
2	Coimbra et al. (2011)	MSC^a^	2:1 HA:CS scaffold / Sigma–Aldrich, Sintra, Portugal / Not mentioned	Cells were seeded with the scaffold; the ratio is not mentioned	MTS assay	Neutral effect
3	Lambricht et al., 2014	SCAP^a^	Tyramine-based hyaluronan hydrogel / Corgel, BioHydrogel Lifecore, Chaska, USA/ Not mentioned	Cells were seeded on the surface of the hydrogel	Live/dead assay	↓Cell viability ↑ Cell activity
					MTS assay	
4	Umemura et al. (2016)	DPSCs^a^,^d^	HA sodium salt / Nacalai Tesque Co., Kyoto, Japan / HMW^d^	10 μg/mL, 20 μg/mL, and 50 μg/mL HA in cell culture medium	MTT assay	Neutral effect
					Immunofluorescence staining of CD44	
					Flow cytometry of CD44	
					Western blotting of CD44	
5	Yang et al. (2016)	Human fibroblasts	Cross-linked HA/HA (Shangdong Freda Biopharm), BDDE (Sigma) / 1,500 kDa	0.5 mg/mL and 1 mg/mL cross-linked HA gels in culture medium	MTT assay	Neutral effect
6	Chrepa et al. (2017)	SCAP^a^	Dermal filler / Restylane, QMed, Uppsala, Sweden / Not mentioned	1:10 culture medium containing SCAP to Restylane ratio placed in confined Transwell inserts	CellTiter-Glo Luminescence assay	↑ Cell viability
7	Cvikl et al. (2017)	primary DPC^a^	Sodium hyaluronate / Regedent, Zurich, Switzerland./ (2 mg of 2.5MDa +16 mg of 1 MDa) mixed^c^	The collagen scaffold was soaked in HA for 1 min	MTT assay	Neutral effect
					BrdU incorporation	
8	Niloy et al. (2020)	DPSC^a^	AEMA–modified HA Hydrogel^d^/ Bloomage Freda Biopharm/ 18-kDa, 270-kDa^c^	0.5%, 1%, and 3% w/v. Hydrogel in cell culture medium.	MTT assay	Neutral effect No toxicity Maintained spherical shape
					Hoechst 33,342 live cell staining	
					Propidium iodide dead cell staining	270 kDa HA Maintained stemness
					qRT-PCR for NANOG and SOX2 of DPSCs	
9	Schmidt et al. (2021)	DPSCs^a^	Pharmaceutical grade HA/Contipro a.s., Dolní Dobrouc, Czech Republic / LMW (800, 1,600, and 15,000 Da)	100 μmol/L HA in culture medium	Cumulative population doubling	Neutral effect
					Expression of CD105 (endoglin), CD117 (cell proliferation, migration), flow cytometry	↓ Cell size, proliferation, and migration gene expression
10	Silva et al. (2021)	HUVECs^a^ and hDPCs	HA modified GelMA Hydrogels^d^/ Sigma-Aldrich + Lifecore / HMW^d^ 1.5–1.8 MDa HA, + LMW^d^ < 10 kDa HA^c^	DPCs were encapsulated in the gel, and HUVEC were seeded onto the surface of the hydrogels.	Metabolic Activity Assessment	↑ Cell proliferation LMW ˃ HMW
					Total amount of double-stranded DNA	
					Immunocytochemistry	
					Confocal microscopy, after staining with CD31 and CD44	
11	Kim et al. (2022)	hDPC	HA-Collagen Hydrogel/ Not mentioned/ Not mentioned	HA-Collagen hydrogel was soaked in the medium for 24 h, and then the medium was used	Cell counting kit-8 kit	Neutral effect
12	AlHowaish et al. (2022b)	hMSC^a^ in 5 mm length root segment of a human permanent tooth	Dermal filler / Restylane, Galderma, Lausanne, Switzerland/Not mentioned	1:1 culture medium containing hMSC to Restylane ratio placed in confined Transwell inserts	AlamarBlue assay	Neutral effect
13	Atila et al. (2022)	DPSC^a^ and HUVEC^a^	Sodium Hyaluronate powder / Hysilk, Contipro, Czech Republic / LMW	Cells were cultured on the surface of hydrogels	Live/dead staining	↑ Cell proliferation
14	Schmidt et al. (2023)	DPSCs^a^	Pharmaceutical grade HA/ Contipro a.s., Dolní Dobrouc, Czech Republic/ LMW; 800 Da, 1,600 Da, 15,000 Da, 237,000 Da, + HMW HA; 1,,500,000 Da	100 μmol/L LMW HA in culture medium 150 mg/100 mL for HMW HA in culture medium	Population doubling (PD) count	Neutral effect
					Cell diameter	Neutral effect
					flow cytometry for CD44 CD45 CD29, CD 73 CD 90	Neutral effect on stemness Neutral effect on cell adhesion and migration
					qPCR CD29, CD44, CD49F, CD73, CD90, and CD105	
15	Dorterler et al. (2024)	L929 mouse fibroblast cells	GelMA-HA hydrogel^d^/ Not mentioned/ Not mentioned	Gel was applied to the cells. The ratio was not mentioned	MTT assay	Neutral effect

^a^ SCAP: stem cells from apical papilla. hDPC: human dental pulp cells. hMSC: human mesenchymal stem cells. DPSC: dental pulp stem cells. HUVEC: human umbilical vein endothelial cells.

^b^ HMW: high molecular weight. LMW: low molecular weight.

^c^ kDa: kilodalton. MDa: mega Dalton = 1 million Dalton.

^d^ HA:CS: hyaluronate:chitosan. AEMA: 2-aminoethyl methacrylate. GelMA: gelatine methacryloyl.

HA can be used in pure or modified forms to enhance its properties for endodontic use. Coimbra et al. (2011) combined HA with chitosan to improve biocompatibility, and Lambricht et al. (2014) studied HA hydrogel combined with tyramine for improved mechanical properties, comparing the modified materials with control groups, and reported no evidence of cytotoxicity. Both studies suggested that these materials are promising candidates for pulp regeneration procedures.

HA is primarily derived from animal and bacterial sources, with its origin affecting endotoxin level, protein residuals and molecular weight (MW). Bacterial-derived HA has lower endotoxins and residual protein content, resulting in higher purity compared to animal-derived HA. The MW of HA from animal sources can reach up to 20,000 kDa, though this can be adjusted through various processing techniques (Snetkov et al., 2020). The majority of articles in this review did not specify the origin of the HA. However, they did reference the production source.

Tziafas et al. applied different concentrations of HA from bovine vitreous humour (grade IV) on dental papillae of mouse embryos. This was the first publication investigating the HA effects on pulp regeneration in 1988. Demonstrated that applying HA to odontoblasts preserved the polarised shape of the cells, which they interpreted as an indication of ongoing production of tubular dentine (Tziafas et al., 1988).

Inuyama et al. (2010) cultured odontoblastic cells on an HA sponge made from rooster combs and evaluated the inflammatory response compared to collagen sponges. They also planted the HA sponge into amputated dental pulp in a rat and conducted histological analysis and PCR evaluation of inflammatory markers (IL-6, TNF-α, and GAPDH) after one and 3 weeks. Both collagen and HA sponges induced pulp regeneration and blood vessel formation. Moreover, the HA sponge better preserved the odontoblastic cell phenotype. In the *in vivo* setting, the inflammatory activity in response to the HA sponge was decreased compared to the collagen scaffold, which contributed to the greater capacity of the HA to suppress the release of the cytokines.

Chen et al. (2016) applied high MW HA derived from *Streptococcus* equi to primary dental pulp cells (DPC) and recommended it as a promising biomaterial for dental pulp regeneration.

#### 4.1.2 HA molecular weight and cross-linking

Since molecular weight (MW) and cross-linking of the HA are critical determinants of its biological and mechanical properties (Snetkov et al., 2020). It is essential to mention the MW values of the HA and the degree of crosslinking for a clearer interpretation of the results. Unfortunately, this information is missing in many of the included articles. Moreover, there is no universal agreement on the definition of high and low MW (Schmidt et al., 2023). Some define low MW as 10–500 kDa and high MW as over 500 kDa (Chistyakov et al., 2019), while others classify MW into low (<1,200 kDa), medium (1,200–3,600 kDa), high (3,600–10,000 kDa), and ultra-high (>10,000 kDa) (Wu et al., 2021). It is therefore an ambiguous practice to write high or low MW without specifying the exact value.

Four articles compared between low MW and high MW HA. Schmidt et al. published two studies investigating the effects of HA with different molecular weights on DPSC (Schmidt et al., 2021; Schmidt et al., 2023), where HA was added to the cell medium culture directly. In their 2021 study, they used HA with low MW values of 800, 1,600, and 15,000 Da. While none of the HA types exhibited cytotoxic effects, all reduced cell size and migration rates compared to the control. In their 2023 study, they repeated the experiment using HMW (1,500 kDa) and low MW (800 Da, 1,600 Da, 15 kDa, 237 kDa) of HA. They concluded that HA, regardless of its MW, has a neutral effect on cell migration and adhesion, while maintaining DPSC stemness. They attributed the variability in results to the high heterogeneity, which might have influenced the response to HA (Gronthos et al., 2000; Gallo et al., 2025).

Silva et al. (2021) reported that low MW (<10 kDa) HA presented better results in terms of proliferation and expression of angiogenic markers (KDR and CD31) of hDPC compared to high MW HA (1.5–1.8 MDa).

While Niloy et al. (2020) examined the effect of varied MW of a commercial HA hydrogel (18 kDa and 270 kDa), modified with aminoethyl methacrylate, on proliferation, morphology and stemness of DPSC. The study concluded that this combination preserved the spherical morphology and stemness of the cells, without compromising cell viability, in comparison to two-dimensional cell cultures. They found that higher MW HA (270 kDa) showed better stemness preservation compared to low MW HA (18 kDa). They attributed these results to the slower degradation rate of high MW HA compared to low MW HA, which maintains its structural integrity for longer periods. This prolonged stability provides support for cells, promoting the maintenance of stemness and viability.

The results of Silva et al. and Niloy et al. cannot be directly compared due to differences in materials and methodologies. Silva et al. seeded cells onto the surface of HA-modified GelMA Hydrogels, while Niloy et al. seeded cells in a culture medium mixed with AEMA–modified HA Hydrogel.

Among the included *in vitro* articles, three strategies for adding HA to cells were observed, depending on the viscosity of HA. The first strategy involved using liquid HA, which was added directly to the cell culture media. The second strategy placed HA sponges or high MW gels at the bottom of the dishes, with cells seeded on top. The third strategy was to soak the HA hydrogel in a cell culture medium, which was then used to culture the cells. These methodological differences directly affect the geometry of the cellular arrangement and hence interactions with the biomolecules.

The higher the concentration of the hydrogel, the longer it can maintain its structural integrity and, consequently, its influence on cell behaviour (Burdick et al., 2004). High HA concentration resulted in a gel-like consistency with increased viscosity, whereas low concentrations of HA produced a liquid-like consistency with lower viscosity. Accordingly, high viscosity HA supports the formation of a 3D cell culture, whereas low viscosity HA forms a 2D cell culture. The methods of HA addition varied across most articles, likely due to the diversity in experimental protocols among the various research centres conducting these studies.

Tziafas et al. evaluated the effect of different concentrations of HA (0.1, 0.2, and 0.4 mg/mL) and found that concentrations of 0.1 and 0.2 mg/mL preserved the polarised shape of the odontoblast as a sign of active odontogenesis. Niloy et al. tested the effect of three HA concentrations (0.5%, 1%, and 3% w/v) on DPSC, and all concentrations showed no cytotoxicity. Umemura et al. tested different HA concentrations (10 μg/mL, 20 μg/mL, and 50 μg/mL) and found that dentine mineralisation of DPSC increased with higher HA concentrations.

Bagio et al. and Nugraheni et al., in 2023, studied the effects of HA on dentinogenic differentiation of the DPSCs. Apparently, they performed their research in the same research centre at the University of Malaysia. They both used High MW (3 MDa) dermal filler, which was mixed with the cell culture medium in different concentrations (10 μg/mL, 20 μg/mL and 30 μg/mL). Investigations of ELISA for DSPP expression on days 7 and 14, and Alizarin red were employed by Nugraheni et al. While investigations of Flowcytometry of TGF β1 expression, and ELISA for CD44 were employed by Bagio et al. They both concluded that HA of different concentrations enhanced the ability of the cells to differentiate into new dentine, especially the concentration of 30 μg/mL HA, which showed the highest results.

We can notice that a higher concentration of HA [50 μg/mL (Umemura et al., 2016), and 30 μg/mL (Bagio et al., 2023; Nugraheni et al., 2023)] lead to better results in supporting DPSC. This could be due to the high viscosity of the medium, which provides an environment closer to the natural extracellular matrix.

### 4.2 Role of HA in cell viability, proliferation, and stemness in the dental pulp regeneration context, *in vitro*

Cell viability, cell proliferation, and stemness refer to different cell properties. Viability indicates that the cells are alive, but it does not necessarily imply their ability to proliferate into daughter cells. On the other hand, cell stemness refers to the capacity of the cells to self-renew and produce differentiated progeny (Mullard, 2008). All these properties are important in the endodontic regeneration process. In this review, all included studies agreed that HA does not affect cell viability, a consistent finding even in non-dental applications of HA (Saravanakumar et al., 2022). Notably, Chrepa et al. mentioned that HA increased cell viability compared to the control groups (Chrepa et al., 2017). This highlights HA’s potential as a highly biocompatible natural scaffold, being a component of the extracellular matrix (An et al., 2023).

Dorterler et al. (2024), mixed the GelMA hydrogel with HA and examined its cytotoxicity and antimicrobial properties. They compared GelMA/HA to hydroxyapatite and silver nanoparticles. All the study groups showed no cytotoxicity using the MTT assay. And HA-modified-GelMA showed the highest ability to provide antimicrobial activity. The authors recommended it as a suitable biomaterial for endodontic regeneration due to its biocompatibility and antimicrobial effect.

Most studies reported that cell proliferation was not affected by the addition of HA (Table 6). However, Atila et al. and Silva et al. observed increased cell proliferation in the HA-treated groups. Lambricht et al., using a tyramine-based HA hydrogel, found that while this modified hydrogel negatively affected cell viability over the long term, it promoted cell proliferation. They attributed these findings to the complex interactions between the hydrogel and cells, including the biodegradation of HA over time, which alters pore size, nutrient availability, and the structural integrity of the environment.

Cvikl et al. (2017) soaked a collagen scaffold in HA for 1 minute before applying it to the primary dental pulp cell culture, and compared it to a collagen scaffold without HA. Kim et al. (2022) also used collagen scaffolds with HA; however, in this research, they soaked collagen and HA in a culture medium before using this medium to cultivate human DPCs. Both studies found that HA did not impact cell viability and used collagen with HA for its superior mechanical integrity.

Researchers used different techniques to test cell viability and proliferation, like MTT, MTS, CellTiter-Glo Luminescence assay, cell counting kit, AlamarBlue assay, BrdU incorporation, Cumulative population doubling, and live/dead count kit. Flow cytometry was used to analyse markers such as CD29, CD44, CD45, CD73, CD90, CD105, and CD117, while Western blotting was used for CD44, metabolic activity assessment. qPCR was also utilised to measure gene expression of markers like CD29, CD44, CD49F, CD73, CD90, CD105, and CD117. The wide range of testing methods employed makes direct comparison between the articles difficult.

### 4.3 Role of HA in dentin regeneration, *in vitro*

Half of the included studies utilised alkaline phosphatase (ALP) assay as an indicator for dentinogenic induction. ALP is an enzyme secreted during dentine mineralisation, and its activity serves as a marker for this process. Proteins such as DMP-1, DSP, DSPP, and Col1 also increase during dentin formation. The majority of the studies agree that the addition of HA to various cell types—including SCAP, MSC, DPSC, and odontoblastic cells results in enhanced induction of dentinogenesis (Table 7).

**TABLE 7:** Effects of hyaluronic acid on dentinogenic differentiation in cell culture studies.

	Author/year	Cell type	HA form/Source/MW^b^	HA concentration	Dentinogenic assay	Findings
1	Inuyama et al. (2010)	Odontoblastic cell line (KN-3 cells)	HA sponge/ Seikagaku Corporation, Tokyo, Japan/ HMW, 900 kDa	60 µL containing 1^a^10^6 cells were dropped onto each piece of sponge (77 mm^3^)	RT-PCR for IL-6 and TNF-α expressions SEM photographs	No effects on inflammation Maintained the phenotype of KN-3 cells
2	Chen et al. (2016)	Primary DPC	HA from *Streptococcus* equi / Sigma-Aldrich/ HMW	2 mg/mL HA in cell culture medium	ALP assay	↑ Early ALP activity (3 days) ↓ Late ALP activity (14 days)
					Alizarin red S	↑ Dentinogenesis
3	Umemura et al. (2016)	DPSCs	HA sodium salt/ Nacalai Tesque Co., Kyoto, Japan/ HMW	10 μg/mL, 20 μg/mL, 50 μg/mL HA in cell culture medium	Immunofluorescence staining of CD44	↑ Dentinogenesis
					Flow cytometry of CD44	
					Western blotting of CD44	
					ALP assay	
					qRT-PCR for DMP-1 and DSPP gene expression	
4	Chrepa et al.(2017)	SCAP^a^	HA dermal filler/ Restylane, QMed, Uppsala, Sweden/ Not mentioned	1:10 culture medium containing SCAP to Restylane ratio placed in confined Transwell inserts	ALP activity	↑ Dentinogenesis
					qRT-PCR analysis of DSPP, DMP-1, and MEPE genes	
5	AlHowaish et al. (2022a)	hMSC in 5 mm length root segment of a human permanent tooth	HA dermal filler/Restylane, Galderma, Lausanne, Switzerland/Not mentioned	1:1 culture medium containing hMSC to Restylane ratio placed in confined Transwell inserts	ALP activity assay	↑ Dentinogenesis
					RT-PCR of DSPP gene expression	
6	Atila et al. (2022)	DPSC	Sodium Hyaluronate powder/ Hysilk, Contipro, Czech Republic / LMW	Cells were cultured on the surface of hydrogels	PCR for COL1A1, ALP, OCN, Axin-2, DSPP, and DMP1 gene expression	↑ Dentinogenesis
7	Bagio et al. (2023)	hDPSCs	Dermal filler / Z fill deep^®^, New-Ulm, Germany / HMW 3 MDa	10 μg/mL, 20 μg/mL, and 30 μg/mL HA in cell culture medium	Flowcytometry of TGF-β1 expression	↑ Dentinogenesis 30 μg/mL highest group
					ELISA for CD44	
					Alizarin red	
8	Nugraheni et al. (2023)	hDPSCs	Dermal filler / Z Fill Deep^®^, New-Ulm, Germany / HMW 3 MDa	10 μg/mL, 20 μg/mL, and 30 μg/mL HA in cell culture medium	ELISA for DSPP expression on days 7 and 14	↑ Dentinogenesis 30 μg/mL highest group
					Alizarin red	

^a^ SCAP: stem cells from apical papilla. hDPC: human dental pulp cells. hMSC: human mesenchymal stem cells. DPSC: dental pulp stem cells. HUVEC: human umbilical vein endothelial cells.

^b^ HMW: high molecular weight. LMW: low molecular weight.

^c^ kDa: kilodalton. MDa: mega Dalton = 1 million Dalton.

Chrepa et al., in 2017 mixed HA dermal filler (Restylane) with the cell culture in a ratio of 1:10 (filler to cell culture medium) (Chrepa et al., 2017). They used SCAP in a 3D cell culture model, with the cells confined between two Transwell inserts to facilitate the medium change and prevent the gel from escaping into the culture medium. In contrast, AlHowaish et al., in 2022, used the same material with a few modifications; using filler at a ratio of 1:1 (filler to culture medium), with hMSC in a 3D cell culture model confined within a 5 mm length root segment. This root segment was placed in a Transwell insert to ensure nutrient supply from above and below the cell culture (AlHowaish et al., 2022a). Despite the slight difference in techniques, both studies observed similarities in the significant increase of cell viability and differentiation markers of ALP activity and DSPP upregulation.

In 2016, Chen et al. investigated the odontogenic potential of Primary DPC, while Umemura et al. (2016) Studied DPSCs proliferation and cellular differentiation induced by CD44 expression, along with the odontogenic differentiation. Chen et al. applied HA in the form of HMW HA at a 2.0 mg/mL concentration on primary dental cells using a pulse treatment method, where HA was administered for 3 days, followed by 18 days of culture in α-minimal essential medium (α-MEM) supplemented. ALP activity and the mineral deposition assessment showed that HA has a positive effect on the cellular dentinogenic differentiation of these cells. In contrast, Umemura et al. utilised HA at concentrations of 10 μg/mL, 20 μg/mL, and 50 μg/mL in the cell culture medium at different time points. Although the MTT assay showed that HA had no significant effect on DPSC proliferation, ALP activity, and the expression of dentinogenic markers such as DMP-1 and DSPP, it confirmed that HA enhanced the dentinogenic differentiation of DPSCs.

### 4.4 Role of HA in Re-vascularisation of dental pulp, *in vitro*

Few studies have investigated HA effects on revascularisation in an endodontic context (Table 8). Most of these studies confirmed that the addition of HA to cell culture (HUVECs and hDPCs) induced angiogenesis markers like MMP-2, MMP-9, VEGFA, VEGFR2, eNOS, and TGF-β1. However, Schmidt et al. (2021) found that the application of low molecular weight HA to DPSCs resulted in a decrease in angiogenic marker expression.

**TABLE 8:** Effects of hyaluronic acid on neovascularisation in cell culture studies.

	Author/Year	Cell type	HA form/Source/MW	HA concentration	Neovascularization assay	Findings
1	Schmidt et al. (2021)	DPSCs	Pharmaceutical grade HA / Contipro a.s., Dolní Dobrouc, Czech Republic / LMW (800, 1,600, and 15,000 Da)	100 μmol/L HA in culture medium	Expression of CD105 (endoglin), CD117 flow cytometry	↓ angiogenic gene expression
2	Silva et al. (2021)	HUVECs and hDPCs	HA-modified GelMA Hydrogels / Sigma-Aldrich + Lifecore / HMW; 1.5–1.8 MDa HA + LMW; <10 kDa HA,	Cells were cultured on the surface of the hydrogels	Zymography for MMP-2 and MMP-9	↑ angiogenic responses in LMW A-HA
3	Atila et al. (2022)	HUVEC	Sodium Hyaluronate powder / Hysilk, Contipro, Czech Republic/ LMW	Cells were cultured on the surface of the hydrogels	PCR for VEGFA, VEGFR2, and eNOS gene expression	↑ Angiogenic gene expression of HUVEC
4	Bagio et al. (2023)	hDPSCs	Dermal filler / Z fill deep^®^, New-Ulm, Germany / HMW 3 MDa	10 μg/mL HA, 20 μg/mL HA and 30 μg/mL HA in cell culture medium	Flowcytometry of TGF-β1 expression	↑ TGF-β1 expression HA 30 μg/mL highest

^a^ SCAP: stem cells from apical papilla. hDPC: human dental pulp cells. hMSC: human mesenchymal stem cells. DPSC: dental pulp stem cells. HUVEC: human umbilical vein endothelial cells.

^b^ HMW: high molecular weight. LMW: low molecular weight.

^c^ kDa: kilodalton. MDa: mega Dalton = 1 million Dalton.

Silva et al., in 2021 were the first to apply HA on human umbilical vein endothelial cells (HUVECs) along with DPSC (Silva et al., 2021). They used commercially available HA-modified GelMA hydrogel. Due to the high viscosity of hydrogel, they culture the cells on the surface of the gel. Angiogenesis was assessed using zymography for MMP-2 and MMP-9, while proliferation was evaluated through metabolic activity assays, DAPI, and phalloidin-stained micrographs. The HA used had two molecular weights: high molecular weight (HMW, 1.5–1.8 MDa) and low molecular weight (LMW, <10 kDa). Their findings indicated that LMW HA promoted greater cell proliferation and angiogenic differentiation compared to HMW HA and control groups.

In 2022, Atila et al. repeated the Silva et al. experiment using low MW HA alone. They seeded the HUVECs on the hydrogel surface, the same as in the previous study (Atila et al., 2022). VEGFA, VEGFR2, and eNOS gene expression were assessed as the markers of angiogenesis. The results demonstrated improved outcomes for the HA groups compared to the negative controls.

### 4.5 Role of HA in neurogenesis of dental pulp, *in vitro*

As for the role of HA in neurogenesis of dental pulp, although HA has been proven to promote the healing of sciatic nerve injury in rats (Yang et al., 2023). And DPSCs have demonstrated the ability to differentiate into neurons (Al-Maswary et al., 2022). No studies have been found that assess the effectiveness of HA on cell neurogenic differentiation in an endodontic context.

### 4.6 The influence of HA on dental pulp regeneration *in vivo*

Few articles have assessed the effectiveness of HA in dental pulp regeneration in animal models (Table 9). These studies evaluated the histological characteristics of dental pulp tissue in animals following exposure to HA. All concluded that HA positively influences pulp tissue regeneration compared to control groups. Sasaki et al. observed reparative dentin bridges of varying thicknesses 2 weeks after direct pulp capping with HA in the molars of female Sprague-Dawley rats, in comparison to calcium hydroxide treatment (Sasaki and Kawamata-Kido, 1995). They used high MW (1,500–2000 kDa) as a solution directly applied to dental pulps. Inuyama et al. placed a high MW (900 kDa) HA sponge on occlusally amputated dental pulps of Wistar-specific pathogen-free rats in comparison to the no-treatment group and collagen sponge group (Inuyama et al., 2010). In the first week, all study groups exhibited vessel formation and infiltration of inflammatory cells into the HA sponges. By the third week, the sponges had biodegraded and were almost completely resorbed. The HA group showed significantly fewer inflammatory cells compared to the control group; however, slight dentin bridge formation was observed in all groups.

**TABLE 9:** Hyaluronic acid effects in dental pulp regeneration in animal models.

	Author/year	Animal model	HA form/Source/MW	HA concentration	HA delivery method	Pulp injury model	Outcome measures^a^	Effects of HA
1	Sasaki et al. (1995)	Rat molar	HA sodium hyaluronate/ NRD 101, Japan Roussel Co., Tokyo/ 1,500–2000 kDa	HA solution directly over the pulp	Direct pulp capping	Pulp exposure	Histological evaluation	↑ Reparative dentine
2	Inuyama et al. (2010)	Rat molar	HA sponge/ Seikagaku Corporation, Tokyo, Japan/ HMW, 900 kDa	The sponge was placed directly on the pulp	Application of HA sponge on exposed pulp	Occlusally Amputated dental pulp	Histological evaluation	↓ Inflammatory cells Neutral effect on vascularisation Neutral effect on dentine bridge formation
3	Palma et al. (2017)	A dog’s immature premolar	HA:SC/ Not mentioned/ Not mentioned	Mixed with blood from the apex	Inserted in the root canal with a plugger	Apically inflamed exposed dental pulp	Histological evaluation	No effect on apical closure ↓ vascularisation
4	AlHowaish et al. (2022b)	A dog’s immature premolar	HA dermal filler/ Restylane, Galderma, Lausanne, Switzerland/ Not mentioned	Mixed with blood from the apex	Injected into the root canals	Complete pulp removal in immature teeth	Histological evaluation	↑ Vascularization ↑ Collagen fibers ↑ Inflammatory cells
5	Hadi et al. (2023)	Rabbit molar	Hyaluronic acid gel / HyaDent BG, 20 mg Na-hyaluronate/ / 1 mL HyaDent, BG/not mentioned	Gel placed directly	Placed in the pulp chamber	Coronal pulp removal	Histological evaluation IHC of VEGF	↑ Pre-dentine bridge ↑ Vascularization ↑ VEGF expression
6	Bektas et al. (2024)	Rat molar	Commercial HA / (Beijing Wisapple Biotech Co., China) + (Gengigel Teething, Ricerfarma, Italy) + (Gengigel Forte, Ricerfarma, Italy)/ 1.6 MDa + unknown + unknown	0.5% and 1% HA in ZOE cement	Placed in the pulp chamber	Coronal pulp removal	Histological evaluation	↑ Odontoblast layer continuity ↑ Pulp cells vitality

^a^ Only measurements that met the criteria of this systematic review are shown in this table.

AlHowaish et al. (2022b) induced bleeding from the apex of immature premolars of sighthound mixed-breed dogs. Then they mixed 1 mL of HA dermal filler with blood inside the canals, followed by coronal restoration. The addition of HA was compared to a no-treatment control group and a blood clot group. After 13 weeks, the radiographic assessment revealed 85% apical closure with no statistical difference from the blood clot group. The apical closure was either by bone or cementum-like tissues. The study found that bone, dentin, and cementum-like tissues were observed inside the root canals, with cementum-like tissue being the predominant hard tissue type. HA induced a significantly higher inflammatory response compared to the blood clot, which the authors attributed to the biodegradation products of HA. They suggested that the inflammation might contribute to enhanced regenerative outcomes. The HA group also showed significantly higher levels of fibrous connective tissue and new vessel formation compared to the other groups. In contrast, the no-treatment group exhibited cessation of apical closure and remnants of necrotic pulp.

Hadi et al. (2023) injected 0.1 mL of HA hydrogel into the pulp chambers of the upper right incisors of rabbits, with the untreated left upper incisors serving as the control group. After 1 week, an inflammatory response and dentine formation were noticed. By the second week, pre-dentine, differentiated odontoblasts and new blood vessels were detected clearly in the HA group, while the control groups showed necrosis signs. Immunohistochemical analysis revealed a significantly higher detection of VEGF in the HA group compared to the control group in the second week.

Bektas et al. (2024) mixed HA in two commercial forms with zinc oxide eugenol (ZOE), a material commonly used in vital pulp therapy. This combination was applied to the exposed pulp of molars in Sprague-Dawley rats, with a ZOE-only group serving as the control. After 30 days, no statistical difference was recorded among groups regarding intracanal calcification. However, the HA-treated groups demonstrated significantly better pulp vitality and continuity of the odontoblast layer compared to the control group.

Palma et al. (2017) utilised the HA-chitosan (HA:CS) scaffold previously tested by Coimbra et al. (2011) who demonstrated neutral effects on MSCs. In their study, Palma et al. compared the HA:CS scaffold to the blood clot method in the endodontic regeneration of apically inflamed immature premolars in beagle dogs. Their findings showed no significant differences in apical closure or mineralised tissue formation along the radicular walls between the two groups. However, the HA: CS scaffold exhibited less vital tissue formation and vascularisation compared to the blood clot group.

Discouraging results were observed when HA was combined with chitosan and compared to the standard blood clot method, as reported by Palma et al. (2017).

### 4.7 Future directions for HA-based dental pulp regeneration

From our perspective, using HA in animal studies at different concentrations and viscosities while comparing it to the standard treatment and no-treatment controls is the most effective approach to assess its efficacy in dental pulp regeneration. It is also recommended that the effectiveness of HA on neurogenesis be tested in *in vitro* settings for dental pulp regeneration purposes.

Given that the majority of included studies indicated that hyaluronic acid (HA) exhibits a neutral effect on cellular proliferation, it is recommended that future experiments combine HA with other biomaterials to potentially achieve improved outcomes in this area.

The importance of reproducibility and minimising risk in animal research is important to make results more reliable and avoid wasting efforts and resources. It is advisable to refer to the customised tools especially designed for each type of study, like ARRIVE and SYRCLE.

### 4.8 Limitations

Systematic reviews usually have inherent limitations (Owens, 2021). The conclusions of this review are affected by the nature of the available articles. As this analysis is based entirely on preclinical data, its direct application to clinical practice is limited. Because of the variations in methodology, like the use of different animal models and different HA formulations, it was not feasible to perform a meta-analysis. Moreover, using the available RoB tools, most of the included articles showed moderate to high RoB, *in vivo* and *in vitro*. It is also important to consider that the body of literature may tend to publish positive results. Consequently, these findings represent a promising initial evidence base that highlights the obvious need for designing future clinical studies.

## 5 Conclusion

The review highlights the promising yet variable outcomes of using HA in dental pulp regeneration. Differences in sources, forms, application techniques, and molecular weights of HA have led to inconsistent results. Future research should focus on standardised comparisons using varying concentrations and viscosities of HA, ensuring reproducibility and minimal risk in animal studies. The use of customised research tools can enhance reliability, ultimately supporting the development of effective regenerative endodontic procedures.

## Data Availability

The original contributions presented in the study are included in the article/supplementary material, further inquiries can be directed to the corresponding author.
